# Reflex Atrioventricular Block

**DOI:** 10.3389/fcvm.2020.00048

**Published:** 2020-04-03

**Authors:** Richard Sutton

**Affiliations:** National Heart & Lung Institute, Imperial College, London, United Kingdom

**Keywords:** atrioventricular block, vasovagal reflex, carotid sinus reflex, cardiac pacing, cardiac conduction system disease, adenosine

## Abstract

Reflex atrioventricular block is well-recorded although it is considered rare. Recent data suggests that it is less rare than has been supposed. It has been shown to occur in both vasovagal and carotid sinus reflexes. It has to be distinguished from paroxysmal atrioventricular block due to ventricular conduction tissue disease. Low chronic adenosine levels combined with adenosine release may mimic reflex atrioventricular block. Explanations of the mechanism of these phenomena have been lacking until the recent past. The relevance of reflex atrioventricular block to clinical decision-making is as a possible indication for pacing the heart with consideration given to the vasodepressor component of the reflex.

## Introduction

**Figure 1:**
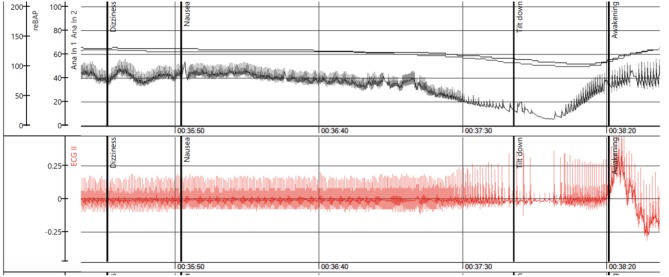
The figure illustrates a positive tilt test in a young female patient. The upper panel shows the beat-to-beat blood pressure recorded by a photoplethysmographic method. The first vertical heavy black line denotes onset of dizziness followed by nausea at the second vertical heavy black line. The third vertical heavy black line denotes tilt down at severe hypotension and loss of consciousness. Rapid recovery follows. The lower panel shows the ECG. There is sinus rhythm until about 20s before tilt down when AVB begins and evolves asystole at tilt down. In recovery there is sinus tachycardia. This is indisputably reflex paroxysmal AVB. Time in minutes and seconds is indicated below (Recording courtesy of A Fedorowski).

Atrioventricular block (AVB) is a well-recognized condition due in most cases either to idiopathic disease of ventricular conduction tissue, the His-Purkinje system, or to ischemic damage to this tissue. Amongst the less common causes are invasion of calcification from calcific aortic valve stenosis and congenital. As AVB became better understood, particularly by employing increasingly sophisticated electrocardiographic monitoring, it became evident that lesser examples of the disease, such as bundle branch block, tended to progress over months or years. Progression often presented evidence of paroxysmal atrioventricular block heralding permanent block. Evidence also emerged in 1980s and 1990s that paroxysmal AVB could be part of carotid sinus or vasovagal reflexes (1–5) (Figure 1).

Reflex AVB, a form of paroxysmal AVB, was less well accepted (1–5) as it may be difficult to distinguish from progression of ventricular conduction system disease (2). More recently, another form of paroxysmal AVB has been attributed to adenosine release in chronic hypoadenosinemia (6, 7). Thus, there are at least three possible pathophysiological mechanisms of paroxysmal AVB.

Conservative thinking concerning reflex AVB has been very strong with a tendency to deny the existence of two of these three mechanisms, namely reflex and hypoadenosinemic types, despite presentation of 4 clear cases of reflex AVB in the Lancet in 1988 (8, 9). Even a recent and very extensive assessment of Danish data in patients of <50 years found that in approximately half of the 1,027 patients receiving a pacemaker in that country between 1996 and 2015 the etiology of AVB was unknown (10) and given the relative youth of the patients reflex causes may have accounted for many. Moreover, they showed that the unknown etiology cases progressively increased in number over the study period.

## Causes and Mechanisms of Paroxysmal Atrioventricular Block

This matter of cause of AVB was particularly illustrated in the opposition of reviewers to publication of the ISSUE (International study of syncope of unknown etiology) 3 study (11) on the basis that the study had only shown a progression of conduction system disease which is expected to respond well to pacing. The authors arguments eventually overcame the objections and the study was published. Nevertheless, at nearly every congress session on this subject the same criticisms arise, i.e., the wrong diagnosis has been made and this is not paroxysmal reflex AVB but progression of ventricular conduction system disease. At the time of publication of the ISSUE 3 (11) and its sub-studies (12, 13) there had been no evidence of progression of AVB in any case, even those that showed complete AVB on insertable loop recorder (ILR) during an episode of syncope.

Analysis of the ILR recordings during syncope in the ISSUE 2 study (4) surprised the investigators, myself included, with the incidence of AVB. Prior to this analysis we had made a classification of rhythm disturbances that occurred on ILR during syncope or symptoms in ISSUE 2 patients (14). This proposal included three types of asystole, Table 1.

**Table 1:** The ISSUE classification of arrhythmias on implantable/insertable ECG loop recorders relating to bradycardia and AVB.

Type 1a: slowing sinus rhythm followed by sinus arrest and ventricular asystole; Type 1b: Progressive sinus bradycardia to <30 bpm followed by AVB with severe bradycardia/asystole; Type 1c: sudden onset of AVB with concomitant increase in sinus rate.

*ISSUE, International study of syncope of unknown etiology; AVB, atrioventicular block*.

The description type 1c raised a question of ventricular conduction tissue disease in the minds of the classification's proposers. Thus, this finding in ~20% of the clinically determined reflex (vasovagal) was unexpected raising the possibility of a different mechanism, namely intrinsic disease of the His-Purkinje system as observed in Stokes-Adams attacks. At this time, even the ISSUE 2 investigators did not completely appreciate that such findings could be compatible with reflex AVB. When the ILR recordings were available from the ISSUE 3 study (8) a very similar finding of type 1c pattern of AVB was made in similar numbers to that seen in ISSUE 2 (4). This consistency was striking but progression to more obvious ventricular conduction system disease was absent in contrast to the ISSUE 1 study of Brignole and colleagues that set out to monitor by ILR patients with bundle branch block finding progression of conduction tissue disease in 42% (15) in 3–15 months of monitoring. In contrast, ISSUE 3 (11) included 2 years of follow-up without such findings where pre-existing conduction system disease was an exclusion from the study.

Following the results of the ISSUE 2 study, the literature again contained definite examples of reflex AVB (3) and evidence also became available for an additional and previously unconsidered form of paroxysmal AVB (6). The latter syndrome was one of “benign” paroxysmal AVB combined with syncope without prodrome, a normal heart and normal ECG but a low adenosine level. It was hypothesized that the low chronic level of adenosine rendered the atrioventricular conduction system very vulnerable to adenosine release precipitating narrow complex AVB. These patients respond very well to pacing. The syndrome of low adenosine AVB has been fully reviewed (7).

Thus, paroxysmal AVB can be considered to be of three types as put forward in the review by Aste and Brignole (16), Table 2.

**Table 2:** Types of paroxysmal atrioventricular block.

1. Reflex AVB (due to vasovagal or carotid sinus mechanisms). 2. Progression of ventricular conduction tissue disease. 3. Hypoadenosinemia.

## Differential Diagnosis

In making a differential diagnosis, these aspects require consideration:

- other associated reflex features e.g., nausea; coincident vasodepression;
- evidence of already existing ventricular conduction tissue disease e.g., bundle branch block;
- plasma adenosine level
- lack of prodrome.

Vasodepression as an integral part of reflex syncope must always be considered because it is always present (17). Not only does it begin much earlier than cardioihibition, by many minutes, but also may be sufficient to drive the blood pressure so low as to cause loss of consciousness before cardioinhibition with AVB occurs (18, 19). It is likely that hypotensive drugs, commonly prescribed in older populations with a hypertensive tendency, actually exaggerate vasodepression. Attention has been given to this in a small trial, STOP-VD, that shows evidence of symptomatic improvement by reduction in hypotensives in a vasovagal syncope, paced group of patients (20).

Unfortunately, adenosine has been a difficult to acquire measurement in routine clinical practice. However, shortly, a simple easily performed test will become available. It has been shown that there are clear differences between vasovagal patients (normal or raised plasma adenosine levels) and those with low adenosine. Carotid sinus syndrome (CSS) patients show similar low adenosine levels to those presenting hypoadenosemic AVB (7). This may explain some differences in behavior between CSS and VVS as yet not fully investigated. It is anticipated that the soon to be greater availability of measurement of plasma adenosine levels and more experience of the lack of progression of conduction tissue disease in reflex AVB compared with already documented, ventricular conduction system disease will clarify this situation.

## Relevance of Cardiac Pacing To Paroxysmal Atrioventricular Block

How do these findings influence a decision to pace the heart? Complete AVB with or without asystole is generally considered an indication for permanent pacing whatever its mechanism. In the case of progression of ventricular conduction system disease the indication to pace is clear when complete AVB or asystole occurs. The relatively new findings reviewed here, reflex and hypoadenosinemic AVB prompt a reassessment of the indications for pacing.

In vasovagal syncope even more than in CSS consideration needs to be given to the timing of bradycardia and timing of loss of consciousness, as vasodepression starts many minutes before cardioinhibition, which might prevent or markedly reduce pacing benefit (17–19). These observations may over-ride that of simply pacing AVB. They may also account for the apparently better results of sensing volume ± contractility of the right ventricle in the closed loop system of Biotronik (Berlin, Germany) rather than simply the onset of bradycardia as in the Rate-drop-response of Medtronic (Minneapolis, MN, USA) (11, 21, 22).

In hypoadenosinemia, the experience is, so far, small but pacing seems to be very effective (23) although it is possible that theophylline treatment could also be effective (24).

## Summary and Conclusions

In summary, reflex atrioventricular block is more common than previously thought as evidenced by the ISSUE 2 and 3 studies (11, 14). It may be an indication to pace but account must be taken of the timing of both bradycardia and loss of consciousness within the reflex episode when making a decision in favor of pacing. Accompanying vasodepression in reflex atrioventricular block also needs consideration as there may be a need to reduce hypotensive medication to improve pacing benefit.

## Author Contributions

The author confirms being the sole contributor of this work and has approved it for publication.

### Conflict of Interest

RS is a consultant to Medtronic Inc., a member of the speakers bureau of Abbott Laboratories Inc. (St. Jude Medical) and stock holder in Edwards LifeSciences Corp., Boston Scientific Inc. and AstraZeneca PLC.
